# Accuracy of dental implant placement using different dynamic navigation and robotic systems: an in vitro study

**DOI:** 10.1038/s41746-024-01178-6

**Published:** 2024-07-06

**Authors:** Zonghe Xu, Lin Zhou, Bin Han, Shuang Wu, Yanjun Xiao, Sihui Zhang, Jiang Chen, Jianbin Guo, Dong Wu

**Affiliations:** 1 Fujian Provincial Engineering Research Center of Oral Biomaterial, School and Hospital of Stomatology, Fujian Medical University, Fuzhou, 350001 China; 2 School and Hospital of Stomatology, Fujian Medical University, Fuzhou, 350001 China; 3 Rhodes College, Memphis, TN USA; 4 Research Center of Dental and Craniofacial Implants, Fujian Medical University, Fuzhou, 350001 China

**Keywords:** Medical research, Outcomes research

## Abstract

Computer-aided implant surgery has undergone continuous development in recent years. In this study, active and passive systems of dynamic navigation were divided into active dynamic navigation system group and passive dynamic navigation system group (ADG and PDG), respectively. Active, passive and semi-active implant robots were divided into active robot group, passive robot group and semi-active robot group (ARG, PRG and SRG), respectively. Each group placed two implants (FDI tooth positions 31 and 36) in a model 12 times. The accuracy of 216 implants in 108 models were analysed. The coronal deviations of ADG, PDG, ARG, PRG and SRG were 0.85 ± 0.17 mm, 1.05 ± 0.42 mm, 0.29 ± 0.15 mm, 0.40 ± 0.16 mm and 0.33 ± 0.14 mm, respectively. The apical deviations of the five groups were 1.11 ± 0.23 mm, 1.07 ± 0.38 mm, 0.29 ± 0.15 mm, 0.50 ± 0.19 mm and 0.36 ± 0.16 mm, respectively. The axial deviations of the five groups were 1.78 ± 0.73°, 1.99 ± 1.20°, 0.61 ± 0.25°, 1.04 ± 0.37° and 0.42 ± 0.18°, respectively. The coronal, apical and axial deviations of ADG were higher than those of ARG, PRG and SRG (all *P* < 0.001). Similarly, the coronal, apical and axial deviations of PDG were higher than those of ARG, PRG, and SRG (all *P* < 0.001). Dynamic and robotic computer-aided implant surgery may show good implant accuracy in vitro. However, the accuracy and stability of implant robots are higher than those of dynamic navigation systems.

## Introduction

The three-dimensional location of dental implants must comply with the principles of biology and mechanics. This is the basis of oral implant therapy^1,2^. An ideal three-dimensional location not only avoids intra-operative damage to important anatomical structures but also facilitates the long-term masticatory and aesthetic performance of the implant^3,4^. In this regard, advances in computer-aided implant surgery (CAIS) have received widespread attention^5–7^. Static computer-aided implant surgery (s-CAIS) and dynamic computer-aided implant surgery (d-CAIS) can be used for osteotomy and implant placement without relying entirely on the dentist’s experience^8,9^. They are associated with improved safety and accuracy of implant surgery. In recent years, robotic computer-aided implant surgery (r-CAIS) has further reduced the need for dental implant experience^10^.

Dynamic navigation systems can be categorised as active or passive based on the mechanism of signal transmission^11^. The optical tracker in an active dynamic navigation system captures infrared light emitted actively by the signal source, while the optical tracker in a passive dynamic navigation system captures infrared light reflected passively by the signal source^12,13^. Implant robots can be categorised as active, passive, semi-active (e.g., co-operated) or tele-operated based on the interaction between the dentist/oral and maxillofacial surgeon and the robotic system^14–16^. The robotic arm of a passive implant robot is not automated. It requires traction and assistance from the dentist/oral and maxillofacial surgeon^17^. Semi-active implant robots used for osteotomy and implant placement operate independently of the dentist/oral and maxillofacial surgeon; however, their robotic arms need to be pulled by the dentist/oral and maxillofacial surgeon to enter and exit the patient’s mouth^18^. Active implant robots can autonomously perform preplanned operations, including the movement of the robotic arm in and out of the patient’s mouth. The surgeon is only responsible for changing the surgical instruments, providing instructions and supervising the robot’s operation^19^.

Both d-CAIS and r-CAIS are associated with improved safety and accuracy of implant surgery compared to freehand implant surgery and s-CAIS^19–22^. In addition to traditional implant surgery, d-CAIS has been used for dental implantation on edentulous jaw and zygomatic bone^23,24^. Case reports of immediate implant placement and lateral and transalveolar maxillary sinus floor elevation in d-CAIS have also been published^25–27^. Implant robots have emerged as a promising CAIS technique in recent years, and a large number of studies have analysed their accuracy in restoring defective dentition, edentulous jaws, and placing zygomatic implant. However, no study has explored and analysed the differences in accuracy between dynamic navigation systems under varying signal transmission and implant robots with a wide range of human-robot interactions^18,28,29^. Therefore, this in vitro study analysed and evaluated the implant accuracy of active and passive dynamic navigation systems and active, passive and semi-active implant robots. Moreover, the effects of the number of implant surgeries on the accuracy of dynamic navigation systems and implant robots were compared. It was hoped that this study would provide a reference for the clinical application of d-CAIS and r-CAIS.

## Results

**Fig. 1:**
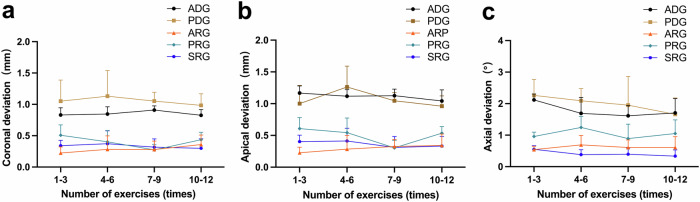
Deviation of four exercise courses for five groups. **a** Coronal deviation, (**b**) apical deviation, (**c**) axial deviation.

A total of 216 implants in 108 models were analysed. Surgeons 1, 2 and 3 successfully placed 144 implants in 72 models with active and passive dynamic navigation systems (ADG and PDG), and surgeon 2 operated active, passive and semi-active implant robots and successfully placed 72 implants in 36 models (ARG, PRG and SRG) (Table 1, Table 2). The results of Kolmogorov-Smirnov and Shapiro-Wilk tests revealed that the data for each group were normally distributed. The 12 implant surgeries were divided into four courses of exercise, and line graphs were plotted based on the implant accuracy of the various dynamic navigation and robotic systems during each exercise (Fig. 1).

**Table 1:** Description with the cohort of surgeons

Surgeon	Professional experience (yrs)	No. of implant surgeries (times)	No. of implants placed (pcs)	Surgical approaches
**1**	>1	24	48	Active and passive dynamic computer-aided implant surgeries
**2**	>5	24	48	Active and passive dynamic computer-aided implant surgeries
**3**	>10	24	48	Active and passive dynamic computer-aided implant surgeries
**2**	>5	36	72	Active, passive, semi-active robotic computer-aided implant surgeries

**Table 2:** Deviations between the planned and placed implants in terms of coronal (mm), apical (mm) and axial (°) deviations of five groups

	Group	No. of surgeon(s)	No. of models	No. of implants	Mean ± SD	95% CI	Min-Max	*P* value^a^	Multiple comparison^b^
Coronal deviation (mm)	ADG	3	36	72	0.85 ± 0.17	0.81–0.89	0.43–1.21	<0.001	ADG < PDG (0.003) ARG < ADG (<0.001) PRG < ADG (<0.001) SRG < ADG (<0.001) ARG < PDG (<0.001) PRG < PDG (<0.001) SRG < PDG (<0.001)
	PDG	3	36	72	1.05 ± 0.42	0.96–1.15	0.12–2.30		
	ARG	1	12	24	0.29 ± 0.15	0.22–0.35	0.05–0.55		
	PRG	1	12	24	0.40 ± 0.16	0.34–0.47	0.16–0.73		
	SRG	1	12	24	0.33 ± 0.14	0.27–0.39	0.10–0.69		
Apical deviation (mm)	ADG	3	36	72	1.11 ± 0.23	1.06–1.17	0.49–1.63	<0.001	ARG < ADG (<0.001) PRG < ADG (<0.001) SRG < ADG (<0.001) ARG < PDG (<0.001) PRG < PDG (<0.001) SRG < PDG (<0.001) ARG < PRG (0.002)
	PDG	3	36	72	1.07 ± 0.38	0.98–1.16	0.40–2.08		
	ARG	1	12	24	0.29 ± 0.15	0.23–0.36	0.06–0.56		
	PRG	1	12	24	0.50 ± 0.19	0.42–0.58	0.13–0.95		
	SRG	1	12	24	0.36 ± 0.16	0.30–0.43	0.07–0.71		
Axial deviation (°)	ADG	3	36	72	1.78 ± 0.73	1.61–1.95	0.54–3.27	<0.001	ARG < ADG (<0.001) PRG < ADG (<0.001) SRG < ADG (<0.001) ARG < PDG (<0.001) PRG < PDG (<0.001) SRG < PDG (<0.001) ARG < PRG (<0.001) SRG < PRG (<0.001) SRG < ARG (0.034)
	PDG	3	36	72	1.99 ± 1.20	1.71–2.27	0.31–4.70		
	ARG	1	12	24	0.61 ± 0.25	0.51–0.72	0.09–1.09		
	PRG	1	12	24	1.04 ± 0.37	0.88–1.19	0.38–1.82		
	SRG	1	12	24	0.42 ± 0.18	0.34–0.49	0.05–0.69		

*ADG* the active dynamic navigation, *PDG* the passive dynamic navigation, *ARG* the active robot, *PRG* the passive robot, *SRG* the semi-active robot, *SD* standard deviation, *CI* confidence interval, *Min* minimum, *Max* maximum.

^a^One-way ANOVA (*P* < 0.05).

^b^Bonferroni method was used to compare the multiple means for homogeneity of variance; Tamhane’s T2 test was used to compare the multiple

means for heterogeneity of variance.

**Fig. 2:**
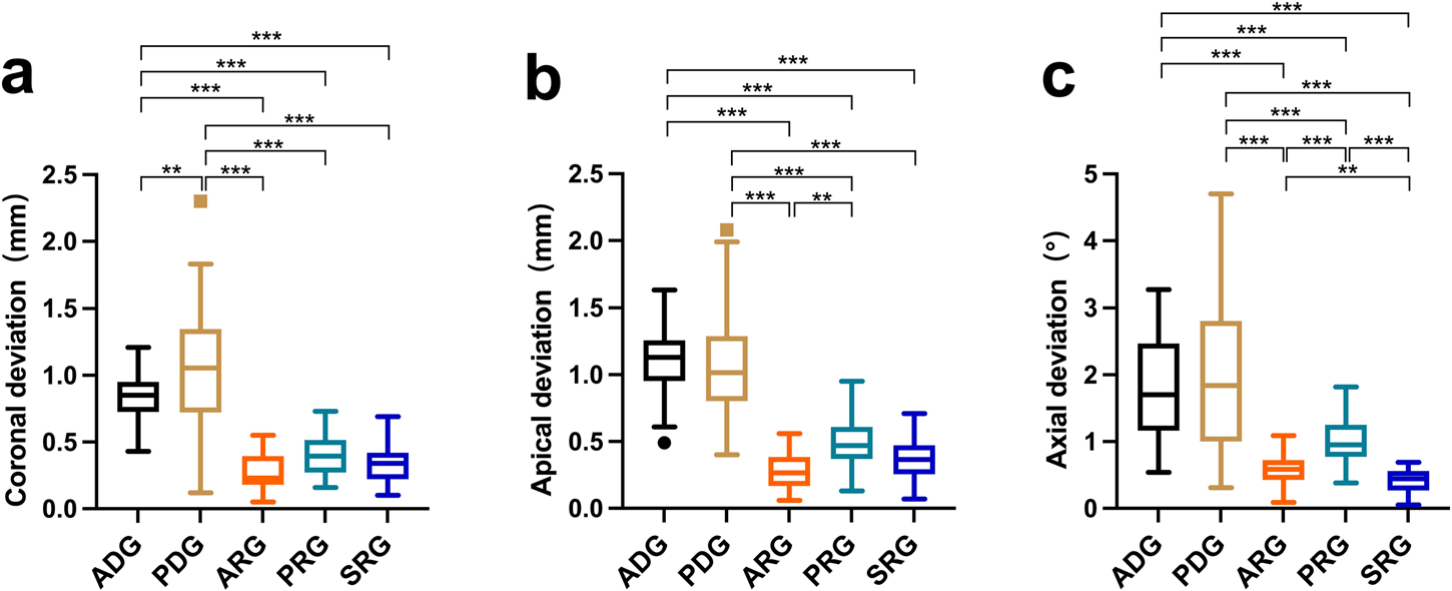
Box plots showing the median, quartile, and range values for the deviations between the planned and placed implants. **a** Box plot of the coronal deviation, (**b**) Box plots of the apical deviation, (**c**) Box plots of the axial deviation.

The coronal deviations of ADG, PDG, ARG, PRG and SRG were 0.85 ± 0.17 mm, 1.05 ± 0.42 mm, 0.29 ± 0.15 mm, 0.40 ± 0.16 mm and 0.33 ± 0.14 mm, respectively. The apical deviations of the five groups were 1.11 ± 0.23 mm, 1.07 ± 0.38 mm, 0.29 ± 0.15 mm, 0.50 ± 0.19 mm and 0.36 ± 0.16 mm, respectively. The axial deviations of the five groups were 1.78 ± 0.73°, 1.99 ± 1.20°, 0.61 ± 0.25°, 1.04 ± 0.37° and 0.42 ± 0.18°, respectively (Table 2). The coronal deviations of ADG and PDG were statistically different (*P* = 0.003). The coronal, apical and axial deviations of ADG and ARG, ADG and PRG, ADG and SRG, PDG and ARG, PDG and PRG, and PDG and SRG differed significantly (all *P* < 0.001). The apical deviations of ARG and PRG were significantly different (*P* = 0.002). The axial deviations of ARG and PRG, PRG and SRG, and ARG and SRG were significantly different (all *P* < 0.001) (Table 2, Fig. 2).

Spearman’s correlation analysis showed that the coronal deviations of the various dynamic navigation and robotic systems (ADG, PDG, ARG, PRG and SRG) were not correlated with the number of implant surgeries (r = 0.064, *P* = 0.767; r = -0.084, *P* = 0.697; r = –0.224, *P* = 0.292; r = –0.216, *P* = 0.312; r = 0.309, *P* = 0.141), the apical deviations of ADG, PDG, ARG, PRG and SRG were not correlated with the number of implant surgeries (r = –0.223, *P* = 0.296; r = –0.119, *P* = 0.581; r = –0.312, *P* = 0.137; r = –0.179, *P* = 0.403; r = 0.327, *P* = 0.119), and the axial deviations of ADG, PDG, ARG, PRG and SRG were not correlated with the number of implant surgeries (r = –0.321, *P* = 0.126; r = –0.347, *P* = 0.097; r = –0.318, *P* = 0.130; r = –0.108, *P* = 0.615; r = 0.038, *P* = 0.862).

Repeated-measures ANOVA showed that none of the exercise effects of coronal, apical and axial deviations were statistically significant (*P* = 0.795, *P* = 0.173, and *P* = 0.078, respectively), indicating that there was no effect of the number of implant surgeries on the implant accuracy of the five groups during the in vitro experiment. However, the between-group effects for the coronal, apical and axial deviations were statistically significant (all *P* < 0.001). This indicates the presence of significant differences in implant accuracy among the five groups. Moreover, the interaction effects on the coronal, apical and axial deviations were not statistically significant (*P* = 0.699, *P* = 0.165, and *P* = 0.362, respectively), indicating that the effect of the number of implant surgeries on accuracy was not significantly different among the five groups (Table 3).

**Table 3:** Repeated-measure analysis of variance (ANOVA) of coronal (mm), apical (mm) and axial (°) deviations between the planned and placed implants

	Exercise effects		Between-group effects		Interaction effects	
	F	*P*	F	*P*	F	*P*
Coronal deviation (mm)	0.230	0.795	178.571	<0.001	0.688	0.699
Apical deviation (mm)	1.793	0.173	167.235	<0.001	1.519	0.165
Axial deviation (°)	2.360	0.078	56.723	<0.001	1.114	0.362

## Discussion

During freehand implant surgery, poor dental operation techniques can damage important anatomical structures such as the maxillary sinus mucosa, inferior alveolar nerve, and blood vessels and nerves in the sublingual space^30,31^. In addition, due to the narrow oral space and the obstruction of oral soft and hard tissues, dentists/oral and maxillofacial surgeons may not be able to complete implant surgery under direct vision^32^. Therefore, in order to improve the accuracy of implant surgery and reduce the related complications, digital technology has been increasingly utilised in implant treatment^20,33–35^. At the end of the 1990s, s-CAIS, derived from freehand surgery, was employed for implant surgery^36,37^. However, s-CAIS is limited by its increased treatment duration due to guide fabrication, increased implant deviation due to guide displacement, and insufficient cooling of the surgical area due to guide placement^38–41^. The limitations of s-CAIS have been addressed by d-CAIS^20,42^. Advances in industrial robotics and the rapid popularisation of 3D imaging technology in the medical field have led to the successful utilization of oral robots in multiple fields, including prosthodontics, orthodontics, implants, endodontics, and oral and maxillofacial surgery^43–45^.

Studies suggest that d-CAIS and r-CAIS will further improve the accuracy of implant surgery while accelerating the digital applications of oral implants^19,46–48^. D-CAIS and r-CAIS have been successfully used to implant placement on the edentulous jaw and zygomatic bone^18,23,24,49^. Studies have also compared the implant accuracy of d-CAIS and r-CAIS^50,51^. However, no studies have compared the implant accuracy of dynamic navigation systems with different signal source transmissions and implant robots with different human-robot interactions in the same experiment. To the best of our knowledge, this experiment is the first in vitro study investigating the implant accuracy of multiple dynamic navigation systems and implant robots. Further, this study also evaluated the effect of the number of implant surgeries on the accuracy of d-CAIS and r-CAIS.

The results of this study revealed that all three groups of r-CAIS exhibited lower coronal, apical and axial deviations compared with d-CAIS due to several factors. First, during the pre-operative preparation, the calibration and registration processes of r-CAIS are achieved by the robotic arm, which is stabler than in d-CAIS. Moreover, the calibration and registration processes involve automatic recognition of the robotic system. The high stability of the robotic arm and the high accuracy of the automatic recognition minimize any deviations of the robotic system^52,53^. Second, implant robots respond to deviations appropriately within milliseconds. The sensitivity of implant robots to deviations and the timeliness of re-planning the implant path enhance the implant accuracy^54^. Finally, subjective factors such as hand-eye coordination, wrist strain and fatigue of the dentist/oral and maxillofacial surgeon may also affect the implant accuracy of d-CAIS^55–57^. When using a dynamic navigation system, dentists/oral and maxillofacial surgeons need to switch back and forth between the operative area and the computer screen. This may lead them to miss critical information and, in turn, contribute to deviations. The high accuracy, efficiency and stability of robots facilitate the accurate and efficient transfer of the 3D position of the implant in the pre-operative planning to the patient’s implant site^58^. Further, compared with humans, robots do not suffer from fatigue and thus, can maintain steady manipulation over a long period of time^59,60^. Ruppin et al. suggested that hand tremors and undetected perceptions contribute to 0.25 mm of lateral deviation and 0.5° of angular deviation^61^. However, the advantages of d-CAIS also need to be stated. For example, when the surgeon holds the implant handpiece to perform osteotomy and implant placement, the surgeon can determine the bone density of the implant site based on the resistance and can adjust the sequence of the drill accordingly in real time^62^. Currently, implant robots cannot display the bone density at the implant site during surgery, which makes it impossible for the surgeon to determine whether or not to modify the implant surgical procedure during r-CAIS.

Ye and Wang et al.^63^ reported that the coronal, apical and axial deviations of a dynamic navigation system at healed single tooth sites were 0.70 ± 0.30 mm, 0.85 ± 0.25 mm and 1.80 ± 0.70 °, respectively. The coronal, apical and axial deviations of the implant robot at healed sites were 0.46 ± 0.29 mm, 0.56 ± 0.30 mm and 1.36 ± 0.54°, respectively. Chen et al. also compared the accuracy of d-CAIS and r-CAIS^50^. The coronal deviations of d-CAIS and r-CAIS were 0.73 ± 0.20 mm and 0.58 ± 0.31 mm, respectively. The apical deviations of d-CAIS and r-CAIS were 0.86 ± 0.33 mm and 0.69 ± 0.28 mm, respectively, and the axial deviations were 2.32 ± 0.71° and 1.08 ± 0.66°, respectively. The accuracies of d-CAIS and r-CAIS in this study are consistent with these and other previous studies^21,51^. However, the aforementioned studies involved only a single type of implant robot or dynamic navigation system. Further, to date, no studies have explored or analysed the effect of the number of implant surgeries on the accuracy of d-CAIS and r-CAIS. The strength of this study lies in its exploration of the differences in implant accuracy among the various dynamic navigation and robotic systems.

Spearman’s test revealed that the number of implant surgeries and implant accuracy were not correlated in each group. Repeated-measures ANOVA showed no statistically significant differences for the exercise and interaction effects in five groups (ADG, PDG, ARG, PRG, SRG), and only the between-group effects were statistically significant. This suggests that neither d-CAIS nor r-CAIS exhibited changes in the implant accuracy with increases in the number of implant surgeries. The effect of the number of implant surgeries on implant accuracy did not significantly differ between the five groups. However, the implant accuracy of each group was not equal. The findings of Sun et al. and Block et al.^64–66^ indicated that although d-CAIS was associated with a learning curve, the implant accuracy of d-CAIS remained unchanged after acquiring proficiency in the hardware and software of the dynamic navigation system. Dentists with different implant experiences display similar accuracy after reaching a plateau. The r-CAIS procedure requires surgical instructions and supervision of the robotic operation, which further reduces the role of the human in the accuracy of the implant. Therefore, as long as dentists/oral and maxillofacial surgeons can skillfully operate a robotic system, implant accuracy will not be affected^58^.

Comparison and analysis of the maximum coronal, apical and axial deviations of d-CAIS and r-CAIS is useful in guiding the clinical application of d-CAIS and r-CAIS. In ADG, the maximum coronal, apical and axial deviations were 1.21 mm, 1.63 mm, and 3.27°, respectively. In PDG, the maximum coronal, apical and axial deviations were 2.30 mm, 2.08 mm and 4.70°, respectively. These findings suggest the possibility of occasional large deviations in d-CAIS. As suggested by Somogyi-Ganss^67^, even in d-CAIS, surgical planning should still follow Worthington’s recommendation for a safety distance of 2 mm in the implant area, to ensure adequate safety and avoid damage to important anatomical structures^68^. This study compared the implant accuracy of dynamic navigation systems with different signal source transmissions and implant robots with different human-robot interactions in the same experiment. The results showed that the maximum deviations of r-CAIS in the coronal, apical and axial deviations were significantly less than those of d-CAIS. Thus, the high stability of the robotic arm reduces the variability in surgical deviation. R-CAIS not only provides physical guidance, as compared to s-CAIS, but also provides real-time feedback with d-CAIS. This will be a focus of interest in the field of oral implant surgery in the future. In orthopaedic surgery and neurosurgery, where operating space is limited, the high stability and precision of robotic surgery are also coming into play^35,69^. It will offer confidence for the wider popularization and application of implant robots in the future.

The accuracy of imaging determines the efficacy of d-CAIS and r-CAIS^7,70^. High-quality CBCT data are necessary for pre-operative planning and registration^71^. In this study, all imaging information was acquired by the same operator in the same CBCT machine under similar parameters. This strategy reduces the experimental error and increases the comparability of implant accuracy between different groups.

In this study, the clinical environment was simulated using a head model and resin models. This differs from the actual clinical situation, where the implant accuracy of d-CAIS and r-CAIS may also be affected by factors such as blood, saliva, and oral and head movements^72^. It is unclear to what extent dynamic navigation systems and implant robots would really work in vivo in patients. Further, the resin models used in this study were homogeneous with no differences in the density and hardness of each site^73^. However, the density of patients’ jaws is not homogeneous. Thus, there is a need for future research using animals or cadavers with different jawbone densities.

Further, the registration processes of d-CAIS and r-CAIS can be categorized as invasive or non-invasive^74^. The invasive registration method is mainly utilized in cases of edentulous jaw. All registration procedures in this study were non-invasive and the implant sites were located in the mandible. Studies in the future should investigate whether changes in the registration processes and upper and lower jaws have impacts on the accuracy of d-CAIS and r-CAIS. Another limitation of this study is that both the dynamic navigation systems and the implant robots utilised were from the same country. Future research may wish to utilise systems from different manufacturers and different countries.

Notwithstanding the limitations of this study, it can be concluded that the number of implant surgeries does not impact the accuracy of d-CAIS and r-CAIS when performed by dentists/oral and maxillofacial surgeons proficient in these procedures. Further, d-CAIS and r-CAIS may show good implant accuracy in vitro. However, the accuracy and stability of r-CAIS are higher than that of d-CAIS. With the continuous improvement and optimization of digital technology, r-CAIS appears to be a promising surgical approach. In future studies, additional clinical variables need to be further compared and analysed to verify the accuracy of d-CAIS and r-CAIS.

## Methods

This study was an in vitro model experiment, so the hospital ethics committee waived the ethical approval requirements.

### Study design

Active dynamic navigation system (Dcarer Medical Technology Co., Ltd, Suzhou, China) and passive dynamic navigation system (Dcarer Medical Technology Co., Ltd, Suzhou, China) were divided into two groups: an active dynamic navigation system group and a passive dynamic navigation system group (ADG and PDG), respectively. The active implant robot (Yekebot Technology Co., Ltd., Beijing, China), passive implant robot (Dcarer Medical Technology Co., Ltd, Suzhou, China) and semi-active implant robot (Baihui Weikang Technology Co., Ltd, Beijing, China) were designated as the active robot group, passive robot group and semi-active robot group (ARG, PRG and SRG), respectively. Considering the role of human factors in the implant accuracy of d-CAIS and the high stability of r-CAIS, this study included three surgeons (surgeons 1, 2 and 3) with varying levels of implant experience in ADG and PDG and one surgeon (surgeon 2) in ARG, PRG and SRG. Three surgeons completed 12 implant surgeries in ADG, respectively. Three months later, the same three surgeons completed 12 implant surgery exercises in PDG, respectively. Thus, the three surgeons performed 36 implant surgeries in ADG and PDG, respectively. Each surgeon placed two implants per exercise. The three surgeons were proficient in the hardware and software of dynamic navigation systems and had more than 10, 5 and 1 year of implant experience, respectively. Each of the three groups of implant robots (ARG, PRG and SRG) completed 12 implant surgeries, with two implants in each exercise. The same surgeon (surgeon 2) who had more than 5 years of implant experience assisted with the operation of the implant robot in each group. All surgeons underwent uniform training on models before performing the in vitro study. All understood the whole procedure and the precautions that need to be taken in surgery.

The sample size was determined using the statistical software PASS, version 15.0.5 (NCSS, LLC, Kaysville, United States), with the statistical power and α set to 90% and 0.05, respectively. Based on the implant accuracy of active and passive dynamic navigation systems and active, passive and semi-active implant robots reported in previous studies^11,75^, the minimum sample size required for ADG and PDG was set at 21, and the minimum sample size required for ARG, PRG and SRG was set at 7. Accordingly, the number of implant surgeries performed in this study met these minimum sample size criteria.

### Model preparation and pre-operative planning

**Fig. 3:**
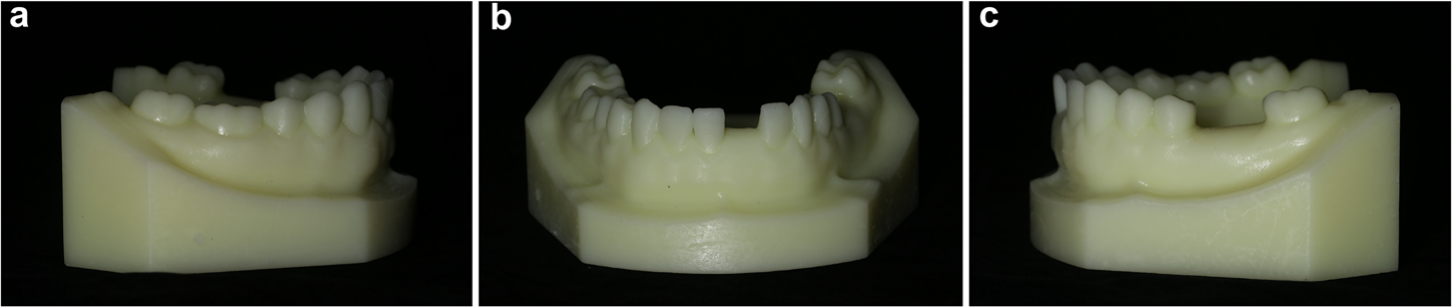
Views of the mandibular model. **a** Right side, (**b**) Front side, (**c**) Left side.

The standard tessellation language (STL) data of the standard dental model (Tuojin Medical Technology Co., Ltd, Foshan, China) was imported into Geomagic Studio, version 2013 (3D Systems Inc., Rock Hill, United States), and the digital model was cut and modified. The model’s mandibular left central incisor and mandibular left first molar were removed in order to simulate dental defects. A total of 108 implant surgeries were completed in this study. Thus, 108 identical mandibular models were three-dimensionally (3D) printed (Wanxiang 3D Technology Co., Ltd, Fuzhou, China) prior to the experiment (Fig. 3).

When scanning the pre-operative cone beam computed tomography (CBCT) of the mandibular models, a registration device containing marker points was fixed to the mandibular model in each group. This facilitated the integration of the pre-operative CBCT, mandibular model and surgical instruments into the same spatial coordinates via point-set registration. The registration device of the active implant robot was designed in the standard tessellation language (STL) data of the mandibular model using DentalNavi navigation software (Yekebot Technology Co., Ltd., Beijing, China) and was later 3D-printed. No imaging information on the registration device was required for its pre-operative CBCT.

The CBCT scans in this study were performed with an i-CAT FLX V10 (KaVo Group, Biberach, Germany) with the following parameters: voltage = 120 kV; tubecurrent = 5.0 μA; focus = 0.5 mm; and voxel size = 0.2 mm. All CBCTs were scanned by the same physician with extensive clinical dental radiography experience.

After scanning, the pre-operative CBCT of each group was imported into a specific software programme for dental implant surgery navigation in the digital imaging and communications in medicine (DICOM) file format. The DICOM images were used to reconstruct the 3D images of the mandibular model before surgery. Two implants (FDI position 31: Nobel PMC 3.5 × 11.5 mm; FDI position 36: Nobel PMC 4.3 × 10 mm) with ideal 3D positions were virtually implanted based on the 3D images of the edentulous regions. The order of drilling and the performance pattern of the osteotomy were standardised for each group during the implant surgery. The mandibular model was fixed on a head model (Tuojin Medical Technology Co., Ltd, Foshan, China) during each implant exercise to simulate regular oral opening, thus realistically simulating clinical scenarios.

### Dynamic navigation-assisted implant surgery

Both active and passive dynamic navigation systems were calibrated and registered before the implant surgery. The purpose of the calibration was to identify and track the real-time location of the implant handpiece (DSG201L, NSK, Nakanishi Inc, Tochigi, Japan) and the mandibular model with the reference plate (Dcarer Medical Technology Co., Ltd, Suzhou, China) worn during implant surgery. During the calibration process, the implant handpiece, reference plate and infrared optical tracker must form an unobstructed straight path. The dynamic navigation system recorded and converted the spatial coordinates of the implant handpiece and the reference plate, thus completing the spatial registration of the implant handpiece and the reference plate. The implant handpiece and the reference plate of the active dynamic navigation system actively emit infrared light to the optical tracker (Polaris Vicra, NDI Inc., Waterloo, Canada), whereas the implant handpiece and the reference plate of the passive dynamic navigation system passively reflect infrared light transmitted by the optical tracker. The registrations of the active and passive dynamic navigation systems were designed to reposition the registration device (Dcarer Medical Technology Co., Ltd, Suzhou, China) to the edentulous regions. The registration process was based on the selection of marker points on the registration device by the implant handpiece, which were matched and registered by the dynamic navigation system to the radiologic marker points in the pre-operative CBCT. At the end of the registration process, the spatial coordinates of the pre-operative CBCT, the mandibular model and the implant handpiece were unified into the same spatial coordinate system.

**Fig. 4:**
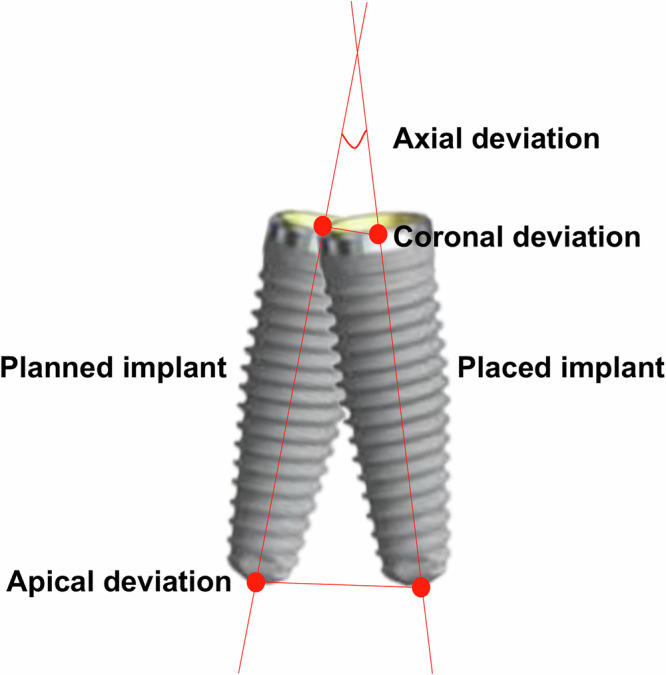
The schematic diagram of measurement accuracy. The deviation between the planned and placed implant was evaluated based on coronal, apical and axial deviations.

After calibration and registration, the computer interface of the dynamic navigation system displayed the position of the drill (Nobel biocare service sag Nobel, Gothenburg, Sweden) in real time in relation to the mandibular model. The three surgeons selectively observed the implant site, implant direction and implant depth in dynamic and static views on the computer interface. The dynamic navigation system prompted the dentist to adjust the 3D position of the implant handpiece in real time based on pre-operative planning. The surgeon then performed osteotomy and implant placement [FDI position 31: Nobel PMC 3.5 × 11.5 mm; FDI position 36: Nobel PMC 4.3 × 10 mm (Nobel biocare service sag, Gothenburg, Sweden)]. Each surgeon repeated the same implant exercise 12 times in the 12 models with the assistance of the active and passive dynamic navigation systems, respectively (Fig. 4a–f). A total of 24 implants were placed by each surgeon in each group. The implant surgeries in ADG and PDG were scheduled at three-month intervals.

### Robot-assisted implant surgery

Similarly, calibration and registration of the active, passive and semi-active implant robots was completed before the surgery. In robotic systems, calibration is intended to enable the optical tracker to recognise and track the real-time position of the robotic arm and the mandibular model with the reference plate worn during the implant surgery. The calibration process of the robotic systems worked with the optical tracker to identify and record the relative spatial position of the reference plate and the robotic arm. An unobstructed straight path between the robotic arm, the reference plate and the optical tracker was also essential. After the calibration process, the mandibular model, pre-operative CBCT, robotic arm and optical tracker were integrated into the same spatial coordinates via maker point-based registration. During the registration process, the marker points of the registration device were automatically recognized by the semi-active implant robotic system, while for the active and passive implant robotic systems, manual selection of the marker points was necessary. Subsequently, the mandibular model and pre-operative CBCT were matched and registered by these marker points. During the implant surgery, the robotic arm accurately recognised and tracked the planned implant in the pre-operative CBCT using the optical tracker. In the active implant robot group, the dentist dragged the implant handpiece fixed at the end of the robotic arm from the outside of the mouth to the implant site before the surgery. The optical tracker simultaneously recorded the process and completed path planning of the robotic arm in and out of the mouth, so as to achieve autonomous movement of the robotic arm of the active implant robot between the outside and inside of the mouth.

During the implant surgery, the robotic arm (UR5, Universal Robots Inc., Odense, Denmark) of the active implant robot moved autonomously from outside the mouth into the edentulous region (5 cm from the starting point of the planned implant site) after the surgeon depressed the pedal control. In contrast, the passive and semi-active implant robots required the surgeon to manually tow the robotic arm to the implant site while depressing the pedal control. When the robotic arms were within the auto-calibration range, each group of implant robots automatically adjusted the position of the handpiece (DSG201L, NSK, Nakanishi Inc., Tochigi, Japan) according to the 3D position of the implant in the pre-operative planning and performed osteotomy along the implant path at a predetermined rate. The active and semi-active implant robots automatically retreated the implant handpiece and robotic arm to the initial intra-oral position after ensuring that the drill (Nobel biocare service sag, Gothenburg, Sweden) had reached the terminal location. The active implant robot then autonomously returned the implant handpiece and robotic arm to their initial extra-oral position. By contrast, the semi-active implant robot required the surgeon to manually tow the arm to its initial position outside the mouth. For the robotic arm of the passive implant robot, assistance was not only required in order to retract the implant handpiece and robotic arm from the implant site to the initial extra-oral position, but manual traction by the surgeon was also required for the robotic arm to perform the osteotomy, place the implant and return to the initial intra-oral position. During the implant surgery using the passive implant robot, the robotic arm only offered 3D physical guidance based on the pre-operative planning. When the 3D position of the implant handpiece did not follow the pre-operative planning, the passive implant robot automatically restricted the implant handpiece to the ideal 3D position. When the implant handpiece was in the ideal 3D position, the robotic arm did not resist the surgeon’s traction. After the robotic arms of each implant robot were retracted to their initial extra-oral position, the surgeon replaced the drill according to the uniform use sequence and performed osteotomy until implants were successfully placed by the robots [FDI position 31: Nobel PMC 3.5 × 11.5 mm; FDI position 36: Nobel PMC 4.3 × 10 mm (Nobel biocare service sag, Gothenburg, Sweden)]. Each group of implant robots repeated the same implant exercise 12 times in 12 models (Fig. 4g-o). A total of 24 implants were placed in each group. The implant surgeries in ADG, PDG and PRG were scheduled at three-month intervals.

**Fig. 5:**
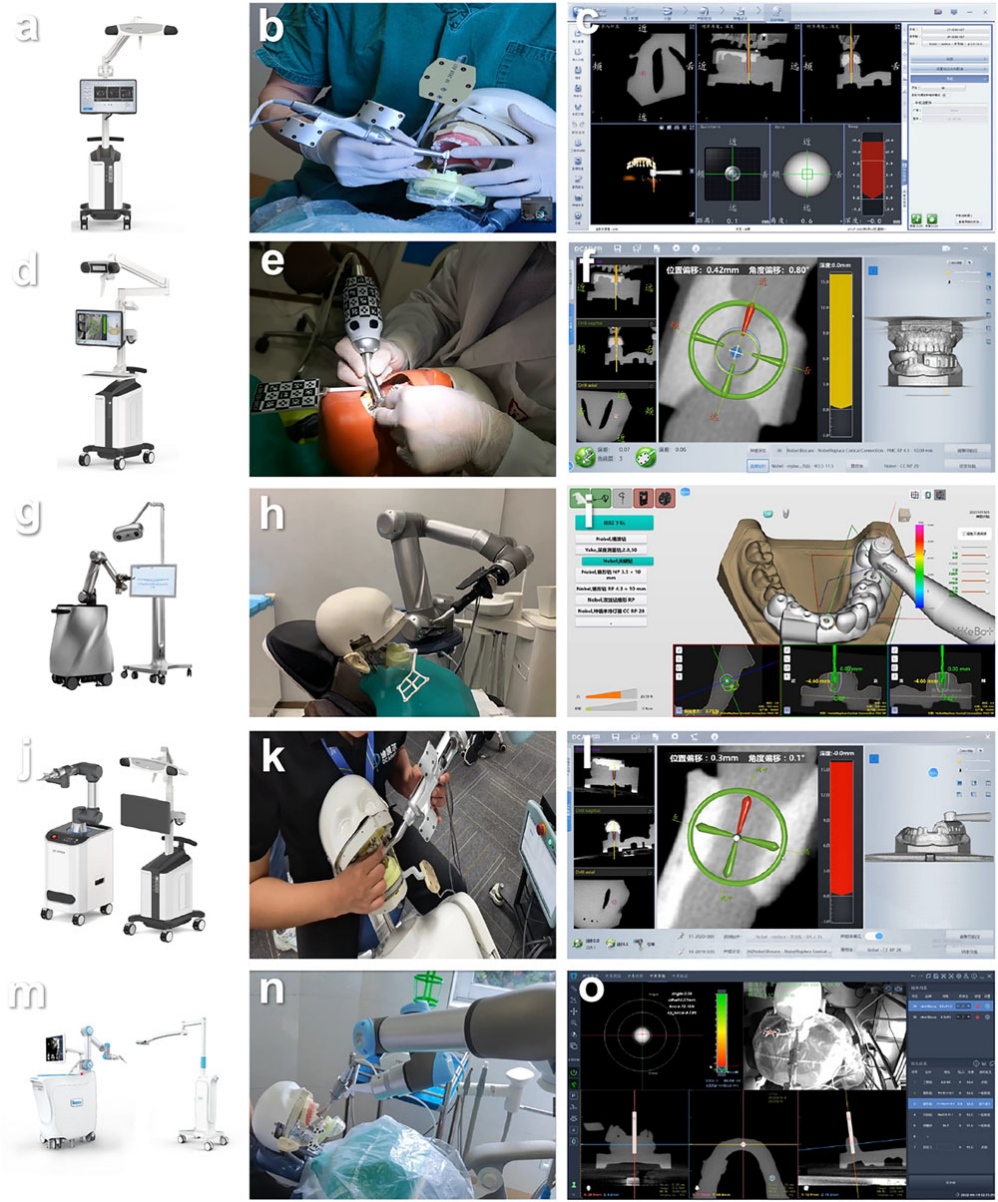
Computer-aided implant surgery and real-time display. **a–c** Active dynamic navigation system, (**d–f**) passive dynamic navigation systems, (**g–i**) active implant robot, (**j–l**) passive implant robot, (**m–o**) Semi-active implant robot.

After two implants were placed in each model, the CBCT was scanned again. The post-operative CBCT and pre-operative planning for ADG, PDG and PRG were imported into the software programme for analysis of the accuracy of the oral implant surgery (Dcarer Medical Technology Co., Ltd, Suzhou, China). Six features shared by the pre-operative planning and post-operative CBCT were randomly selected and integrated. The post-operative CBCT data of the ARG and SRG were imported into the respective surgical navigation software programmes. The points of the registration device or shared features of the pre-operative planning and post-operative CBCT were integrated for registration. The deviation between the pre-operative planning and post-operative CBCT was automatically calculated by each software programme after integration, and the deviation was evaluated based on three indicators (Fig. 5):

1. Coronal deviation: the linear displacement between the placed implant and the planned implant at the centre of the neck platform of the implant (mm).
2. Apical deviation: the linear displacement between the placed implant and the planned implant at the centre of the apical part of the implant (mm).
3. Axial deviation: the intersection angle between the hypothetical central axis of the placed implant and the planned implant (◦).

The same surgeon (B.H.), who was not involved in the implant surgery, was responsible for the integration of the pre-operative planning and the post-operative CBCT. The deviation was determined by the average value obtained after three consecutive integrations.

### Statistical methods

The experimental data were statistically analysed using SPSS 26.0 software (SPSS Inc., Chicago, USA). The descriptive statistical parameters of the five groups were recorded according to the mean, standard deviation, 95% confidence interval and minimum-maximum values. The correlation between the number of implant surgeries and implant accuracy was analysed by Spearman’s test. The normality of the data in each group was evaluated using the Kolmogorov-Smirnov test and Shapiro-Wilk test. Normally distributed data (*P* > 0.05) were then analysed with one-way ANOVA and repeated-measures ANOVA. Non-normally distributed data were analysed with the Kruskal-Wallis H-test. *P* values < 0.05 indicated statistically significant differences. GraphPad Prism8 (GraphPad Software Inc., San Diego, USA) was used to draw the box plots and line graphs.

### Reporting summary

Further information on research design is available in the Nature Research Reporting Summary linked to this article.

### Supplementary information

Deviations between the planned and placed implants of five groups

Reporting Summary

## Data Availability

All data generated or analysed during this study are included in this published article (and its supplementary information files).

## Code availability

The code that supports the findings of this study are available upon reasonable request from the corresponding author.

## References

[1] Gaêta-Araujo H, Oliveira-Santos N, Mancini AXM, Oliveira ML, Oliveira-Santos C (2020). Retrospective assessment of dental implant-related perforations of relevant anatomical structures and inadequate spacing between implants/teeth using cone-beam computed tomography. Clin. Oral. Investig..

[2] Rawal S (2022). Guided innovations: robot-assisted dental implant surgery. J. Prosthet. Dent..

[3] Panchal N, Mahmood L, Retana A, Emery R (2019). Dynamic navigation for dental implant surgery. Oral. Maxillofac. Surg. Clin. North Am..

[4] Hämmerle CHF, Tarnow D (2018). The etiology of hard- and soft-tissue deficiencies at dental implants: A narrative review. J. Periodontol..

[5] Deeb GR, Tran DQ, Deeb JG (2020). Computer-aided planning and placement in implant surgery. Atlas Oral. Maxillofac. Surg. Clin. North Am..

[6] Böse MWH, Beuer F, Schwitalla A, Bruhnke M, Herklotz I (2022). Dynamic navigation for dental implant placement in single-tooth gaps: a preclinical pilot investigation. J. Dent..

[7] Wismeijer D (2018). Group 5 ITI consensus report: Digital technologies. Clin. Oral. Implants Res..

[8] Cecchetti F, Di Girolamo M, Ippolito DG, Baggi L (2020). Computer-guided implant surgery: Analysis of dynamic navigation systems and digital accuracy. J. Biol. Regul. Homeost. Agents.

[9] Bathija, A. et al. Accuracy of static computer-aided implant surgery (S-CAIS) using CAD-CAM surgical templates fabricated from different additive manufacturing technologies. *J. Prosthet. Dent.*10.1016/j.prosdent.2023.03.025 (2023). 10.1016/j.prosdent.2023.03.025 37121851

[10] Fish L (2023). Utilizing robotic technology to place dental implants. J. Oral. Maxillofac. Surg..

[11] Wang XY (2022). The accuracy and learning curve of active and passive dynamic navigation-guided dental implant surgery: an in vitro study. J. Dent..

[12] Zhou Z (2017). Optical surgical instrument tracking system based on the principle of stereo vision. J. Biomed. Opt..

[13] Zhang M (2019). Multiple instruments motion trajectory tracking in optical surgical navigation. Opt. Express.

[14] Wu Y, Wang F, Fan S, Chow JK (2019). Robotics in dental implantology. Oral. Maxillofac. Surg. Clin. North Am..

[15] Yan, Y. & Jia, Y. A review on human comfort factors, measurements, and improvements in human-robot collaboration. *Sensors (Basel)* **22** (2022). 10.3390/s22197431 PMC9572111 36236530

[16] Troccaz J, Dagnino G, Yang GZ (2019). Frontiers of medical robotics: From concept to systems to clinical translation. Annu Rev. Biomed. Eng..

[17] Bolding SL, Reebye UN (2022). Accuracy of haptic robotic guidance of dental implant surgery for completely edentulous arches. J. Prosthet. Dent..

[18] Yang S, Chen J, Li A, Li P, Xu S (2022). Autonomous robotic surgery for immediately loaded implant-supported maxillary full-arch prosthesis: A case report. J. Clin. Med..

[19] Jia, S., Wang, G., Zhao, Y. & Wang, X. Accuracy of an autonomous dental implant robotic system versus static guide-assisted implant surgery: A retrospective clinical study. *J. Prosthet. Dent*. 10.1016/j.prosdent.2023.04.027 (2023). 10.1016/j.prosdent.2023.04.027 37291043

[20] Jorba-García A, González-Barnadas A, Camps-Font O, Figueiredo R, Valmaseda-Castellón E (2021). Accuracy assessment of dynamic computer-aided implant placement: a systematic review and meta-analysis. Clin. Oral. Investig..

[21] Chen W (2023). Accuracy of dental implant placement with a robotic system in partially edentulous patients: A prospective, single-arm clinical trial. Clin. Oral. Implants Res..

[22] Yu X, Tao B, Wang F, Wu Y (2023). Accuracy assessment of dynamic navigation during implant placement: A systematic review and meta-analysis of clinical studies in the last 10 years. J. Dent..

[23] Pozzi A, Hansson L, Carosi P, Arcuri L (2021). Dynamic navigation guided surgery and prosthetics for immediate loading of complete-arch restoration. J. Esthet. Restor. Dent..

[24] Bhalerao A, Marimuthu M, Wahab A, Ayoub A (2023). Dynamic navigation for zygomatic implant placement: A randomized clinical study comparing the flapless versus the conventional approach. J. Dent..

[25] Wei SM (2022). Does machine-vision-assisted dynamic navigation improve the accuracy of digitally planned prosthetically guided immediate implant placement? a randomized controlled trial. Clin. Oral. Implants Res..

[26] Jain S, Solanki A (2021). A dynamic surgical navigational approach for immediate implantation and transcrestal sinus augmentation. J. Indian Soc. Periodontol..

[27] Bishbish O, Kan J, Kim YJ (2023). Guided lateral window osteotomy using dynamic navigation for maxillary sinus augmentation: a novel technique. J. Oral. Implantol..

[28] Yang, S. et al. Accuracy of autonomous robotic surgery for single-tooth implant placement: A case series. *J. Dent*. 10.1016/j.jdent.2023.104451 (2023). 10.1016/j.jdent.2023.104451 36781099

[29] Li C (2023). Autonomous robotic surgery for zygomatic implant placement and immediately loaded implant-supported full-arch prosthesis: a preliminary research. Int J. Implant Dent..

[30] Lin CC (2020). Fully digital workflow for planning static guided implant surgery: A prospective accuracy study. J. Clin. Med..

[31] Chackartchi T, Romanos GE, Parkanyi L, Schwarz F, Sculean A (2022). Reducing errors in guided implant surgery to optimize treatment outcomes. Periodontol 2000.

[32] Naeini EN, Atashkadeh M, De Bruyn H, D’Haese J (2020). Narrative review regarding the applicability, accuracy, and clinical outcome of flapless implant surgery with or without computer guidance. Clin. Implant Dent. Relat. Res..

[33] van Riet TCT (2021). Robot technology in dentistry, part one of a systematic review: Literature characteristics. Dent. Mater..

[34] van Riet TCT (2021). Robot technology in dentistry, part two of a systematic review: An overview of initiatives. Dent. Mater..

[35] Chen YW (2021). Computer-assisted surgery in medical and dental applications. Expert Rev. Med. Devices.

[36] Tahmaseb A, Wu V, Wismeijer D, Coucke W, Evans C (2018). The accuracy of static computer-aided implant surgery: a systematic review and meta-analysis. Clin. Oral. Implants Res..

[37] Di Giacomo GA, Cury PR, de Araujo NS, Sendyk WR, Sendyk CL (2005). Clinical application of stereolithographic surgical guides for implant placement: preliminary results. J. Periodontol..

[38] D’Haese J, Ackhurst J, Wismeijer D, De Bruyn H, Tahmaseb A (2017). Current state of the art of computer-guided implant surgery. Periodontol 2000.

[39] Block MS, Emery RW (2016). Static or dynamic navigation for implant placement-choosing the method of guidance. J. Oral. Maxillofac. Surg..

[40] Pyo SW, Lim YJ, Koo KT, Lee J (2019). Methods used to assess the 3D accuracy of dental implant positions in computer-guided implant placement: A review. J. Clin. Med..

[41] Sigcho López DA, García I, Da Silva Salomao G, Cruz Laganá D (2019). Potential deviation factors affecting stereolithographic surgical guides: a systematic review. Implant Dent..

[42] Papaspyridakos P, De Souza A, Bathija A, Kang K, Chochlidakis K (2021). Complete digital workflow for mandibular full-arch implant rehabilitation in 3 appointments. J. Prosthodont.

[43] He Q (2020). Robotic lateral cervical lymph node dissection via bilateral axillo-breast approach for papillary thyroid carcinoma: a single-center experience of 260 cases. J. Robot Surg..

[44] Wang D (2014). Preliminary study on a miniature laser manipulation robotic device for tooth crown preparation. Int J. Med. Robot.

[45] Ahmad P (2021). Dental robotics: A disruptive technology. Sensors (Basel)..

[46] Pellegrino G (2021). Dynamic navigation in implant dentistry: a systematic review and meta-analysis. Int. J. Oral. Maxillofac. Implants.

[47] Ma F, Sun F, Wei T, Ma Y (2022). Comparison of the accuracy of two different dynamic navigation system registration methods for dental implant placement: A retrospective study. Clin. Implant Dent. Relat. Res.

[48] Bai SZ (2021). Animal experiment on the accuracy of the autonomous dental implant robotic system. Zhonghua Kou Qiang Yi Xue Za Zhi.

[49] Olivetto M, Bettoni J, Testelin S, Lefranc M (2023). Zygomatic implant placement using a robot-assisted flapless protocol: proof of concept. Int J. Oral. Maxillofac. Surg..

[50] Chen J (2023). Comparison the accuracy of a novel implant robot surgery and dynamic navigation system in dental implant surgery: An in vitro pilot study. BMC Oral. Health.

[51] Tao B (2022). Accuracy of dental implant surgery using dynamic navigation and robotic systems: An in vitro study. J. Dent..

[52] Yan B (2022). Optics-guided robotic system for dental implant surgery. Chin. J. Mech. Eng..

[53] Sin M (2023). Development of a real-time 6-DOF motion-tracking system for robotic computer-assisted implant surgery. Sensors (Basel)..

[54] Cheng KJ (2021). Accuracy of dental implant surgery with robotic position feedback and registration algorithm: an in-vitro study. Comput. Biol. Med..

[55] Golob Deeb J (2019). Exploring training dental implant placement using computer-guided implant navigation system for predoctoral students: a pilot study. Eur. J. Dent. Educ..

[56] Pomares-Puig C, Sánchez-Garcés MA, Jorba-García A (2023). Dynamic and static computer-assisted implant surgery for completely edentulous patients. a proof of a concept. J. Dent..

[57] Kalaivani G, Balaji VR, Manikandan D, Rohini G (2020). Expectation and reality of guided implant surgery protocol using computer-assisted static and dynamic navigation system at present scenario: evidence-based literature review. J. Indian Soc. Periodontol..

[58] Zhou LP, Zhang RJ, Sun YW, Zhang L, Shen CL (2021). Accuracy of pedicle screw placement and four other clinical outcomes of robotic guidance technique versus computer-assisted navigation in thoracolumbar surgery: a meta-analysis. World Neurosurg..

[59] Abduo J, Lau D (2021). Accuracy of static computer-assisted implant placement in long span edentulous area by novice implant clinicians: a cross-sectional in vitro study comparing fully-guided, pilot-guided, and freehand implant placement protocols. Clin. Implant Dent. Relat. Res.

[60] Tang T (2019). Factors that influence direction deviation in freehand implant placement. J. Prosthodont..

[61] Ruppin J (2008). Evaluation of the accuracy of three different computer-aided surgery systems in dental implantology: optical tracking vs. stereolithographic splint systems. Clin. Oral. Implants Res..

[62] Xiao Y (2022). Construction of a new automatic grading system for jaw bone mineral density level based on deep learning using cone beam computed tomography. Sci. Rep..

[63] Chen, J. et al. Accuracy of immediate dental implant placement with task-autonomous robotic system and navigation system: an in vitro study. *Clin. Oral Implants Res*. 10.1111/clr.14104 (2023). 10.1111/clr.14104 37248610

[64] Block MS, Emery RW, Lank K, Ryan J (2017). Implant placement accuracy using dynamic navigation. Int. J. Oral. Maxillofac. Implants.

[65] Sun TM, Lan TH, Pan CY, Lee HE (2018). Dental implant navigation system guide the surgery future. Kaohsiung J. Med. Sci..

[66] Sun TM, Lee HE, Lan TH (2019). The influence of dental experience on a dental implant navigation system. BMC Oral. Health.

[67] Somogyi‐Ganss E, Holmes HI, Jokstad A (2015). Accuracy of a novel prototype dynamic computer‐assisted surgery system. Clin. Oral. Implants Res.

[68] Worthington, P. Injury to the inferior alveolar nerve during implant placement: A formula for protection of the patient and clinician. *Int. J. Oral Maxillofac Implants*. **19** (2004). 15508990

[69] De Benedictis A (2017). Robot-assisted procedures in pediatric neurosurgery. Neurosurg. Focus.

[70] Fatemitabar SA, Nikgoo A (2010). Multichannel computed tomography versus cone-beam computed tomography: linear accuracy of in vitro measurements of the maxilla for implant placement. Int. J. Oral. Maxillofac. Implants.

[71] Joda T, Gallucci GO (2015). The virtual patient in dental medicine. Clin. Oral. Implants Res..

[72] Perry S, Bridges SM, Burrow MF (2015). A review of the use of simulation in dental education. Simul. Health..

[73] Dawood A, Marti Marti B, Sauret-Jackson V, Darwood A (2015). 3D printing in dentistry. Br. Dent. J..

[74] Lan K, Tao B, Wang F, Wu Y (2022). Accuracy evaluation of 3D-printed noninvasive adhesive marker for dynamic navigation implant surgery in a maxillary edentulous model: an in vitro study. Med. Eng. Phys..

[75] Xu Z (2023). Accuracy and efficiency of robotic dental implant surgery with different human-robot interactions: An in vitro study. J. Dent..

