# Author Correction: Accuracy of dental implant placement using different dynamic navigation and robotic systems: an in vitro study

**DOI:** 10.1038/s41746-024-01186-6

**Published:** 2024-07-15

**Authors:** Zonghe Xu, Lin Zhou, Bin Han, Shuang Wu, Yanjun Xiao, Sihui Zhang, Jiang Chen, Jianbin Guo, Dong Wu

**Affiliations:** 1 Fujian Provincial Engineering Research Center of Oral Biomaterial, School and Hospital of Stomatology, Fujian Medical University, Fuzhou, 350001 China; 2 School and Hospital of Stomatology, Fujian Medical University, Fuzhou, 350001 China; 3 Rhodes College, Memphis, TN USA; 4 Research Center of Dental and Craniofacial Implants, Fujian Medical University, Fuzhou, 350001 China

**Keywords:** Medical research, Outcomes research

Correction to: *npj Digital Medicine* 10.1038/s41746-024-01178-6, published online 6 July 2024

In this article, the affiliation details in affiliation 1 were incorrectly given as ‘1 Fujian Provincial Engineering Research Center of Oral Biomaterial, Fujian Medical University, Fuzhou 350001, China’ but should have been ‘1 Fujian Provincial Engineering Research Center of Oral Biomaterial, School and Hospital of Stomatology, Fujian Medical University, Fuzhou 350001, China ’. The original article has been corrected.

