# Supplementary material for: Accuracy of dental implant placement using different dynamic navigation and robotic systems: an in vitro study
Source: NPJ Digit Med. 2024 Jul 6;7:182. doi: 10.1038/s41746-024-01178-6 (PMC11227595; doi:10.1038/s41746-024-01178-6)
Supplement: Supplementary file 1 — Deviations between the planned and placed implants of five groups [file 41746_2024_1178_MOESM1_ESM.pdf]

Deviations between the planned and placed implants of five groups

Active dynamic navigation system								
	No. of implant surgeries	Mandibular left central incisor				Mandibular left first molar		
		Coronal deviation （mm）	Apical deviation （mm）	Axial deviation （°）		Coronal deviation （mm）	Apical deviation （mm）	Axial deviation （°）
Surgeons 1	1	0.59	1.09	2.54		1.15	1.63	3.25
	2	0.84	1.32	2.61		0.88	1.25	2.66
	3	0.78	1.24	2.51		0.75	1.10	2.02
	4	0.90	1.29	1.94		0.88	1.27	2.16
	5	0.64	1.04	2.23		0.96	1.26	1.78
	6	1.05	1.59	2.63		1.05	1.31	2.14
	7	0.84	1.18	1.85		0.98	1.21	1.37
	8	0.91	1.10	1.36		0.85	1.01	1.17
	9	0.95	1.15	1.01		0.79	0.90	0.70
	10	0.94	0.95	1.34		0.99	1.21	1.18
	11	0.92	1.06	1.12		1.03	1.18	0.82
	12	0.85	1.04	0.93		0.82	0.96	0.88
Surgeons 2	1	0.64	1.15	2.56		0.70	1.22	2.94
	2	0.66	1.06	1.94		0.79	1.13	2.35
	3	0.84	1.24	2.59		1.21	1.57	2.01

	4	0.67	0.94	1.37		0.85	0.87	0.98
	5	0.58	1.13	2.80		0.95	1.35	2.21
	6	0.84	1.30	2.82		1.06	1.29	1.31
	7	1.07	1.20	1.64		0.94	1.20	1.48
	8	0.92	1.16	1.25		0.95	0.89	0.91
	9	0.85	1.43	3.27		0.96	1.12	1.55
	10	0.84	1.39	2.63		0.72	0.85	1.03
	11	0.43	0.49	2.93		0.85	1.19	1.99
	12	0.74	1.38	3.21		1.19	1.25	1.15
Surgeons 3	1	0.72	0.68	0.92		0.94	0.85	0.54
	2	0.82	1.14	1.64		0.66	1.00	1.90
	3	1.21	1.38	1.70		0.78	0.99	1.44
	4	0.93	1.08	0.71		0.85	0.91	0.57
	5	0.66	0.88	1.21		0.67	0.75	0.75
	6	0.93	1.04	2.14		0.75	0.79	0.66
	7	1.09	1.21	2.07		0.64	0.63	1.70
	8	1.02	1.46	2.60		1.04	1.33	1.70
	9	0.81	1.06	1.91		0.75	1.01	1.54
	10	0.93	0.93	1.99		0.58	0.87	1.66
	11	0.69	0.85	1.13		0.55	1.01	2.80
	12	1.12	1.50	2.70		0.69	0.61	1.16

Passive dynamic navigation system								
	No. of implant surgeries	Mandibular left central incisor				Mandibular left first molar		
		Coronal deviation（mm）	Apical deviation（mm）	Axial deviation（°）		Coronal deviation（mm）	Apical deviation（mm）	Axial deviation（°）
Surgeons 1	1	1.31	0.66	3.11		0.44	0.95	2.83
	2	1.78	1.28	4.31		1.18	0.67	2.23
	3	1.70	1.73	1.81		1.07	1.39	2.06
	4	1.55	1.69	1.24		2.30	1.80	4.21
	5	1.30	1.86	3.27		1.26	1.24	3.78
	6	1.20	1.08	0.63		0.65	0.71	2.67
	7	1.34	1.23	0.78		1.23	1.35	0.84
	8	1.38	1.06	4.70		0.61	1.02	3.65
	9	1.06	0.80	1.71		1.04	1.14	0.41
	10	1.09	0.52	1.87		0.50	0.73	2.59
	11	1.38	0.84	2.04		0.78	1.18	0.69
	12	1.05	1.11	1.94		1.22	1.02	1.12
Surgeons 2	1	0.52	0.66	1.26		0.70	1.07	2.65
	2	1.52	1.50	0.83		0.38	0.40	1.22
	3	1.47	1.30	1.71		1.27	1.03	3.24
	4	0.72	0.94	1.06		1.76	1.48	1.69

	5	0.92	1.18	1.72		0.12	0.46	1.90
	6	0.76	1.29	2.54		0.55	0.55	0.49
	7	0.88	0.81	0.46		0.70	0.77	4.32
	8	0.78	0.85	1.43		0.83	0.91	0.58
	9	0.80	0.90	0.55		0.73	0.77	3.53
	10	0.66	0.61	0.32		0.59	0.65	0.41
	11	0.77	0.61	0.98		0.63	0.66	0.45
	12	1.18	1.33	0.70		1.60	1.72	1.76
Surgeons 3	1	0.33	0.83	3.60		0.88	0.88	2.10
	2	0.64	0.56	1.27		1.07	0.92	0.90
	3	1.26	1.26	2.41		1.38	0.94	3.14
	4	1.51	2.08	3.75		1.49	1.56	1.18
	5	0.90	1.01	2.73		1.83	1.99	1.16
	6	0.84	0.87	2.20		0.72	0.92	1.45
	7	1.01	1.06	0.31		1.57	1.52	0.41
	8	1.60	1.63	2.73		1.53	1.15	2.15
	9	0.85	1.07	1.97		1.04	0.82	4.56
	10	1.35	1.10	2.01		1.33	1.64	1.69
	11	0.49	0.98	3.78		1.16	0.99	1.31
	12	0.77	0.99	3.19		1.14	0.61	3.09

Semi-active implant robot								
	No. of implant surgeries	Mandibular left central incisor				Mandibular left first molar		
		Coronal deviation （mm）	Apical deviation （mm）	Axial deviation （°）		Coronal deviation （mm）	Apical deviation （mm）	Axial deviation （°）
Surgeons 2	1	0.28	0.33	0.47		0.22	0.31	0.41
	2	0.47	0.57	0.68		0.37	0.36	0.50
	3	0.37	0.48	0.69		0.33	0.37	0.56
	4	0.51	0.54	0.35		0.27	0.31	0.30
	5	0.10	0.13	0.25		0.69	0.71	0.45
	6	0.35	0.45	0.67		0.32	0.33	0.27
	7	0.46	0.50	0.59		0.38	0.38	0.09
	8	0.37	0.40	0.52		0.39	0.39	0.56
	9	0.16	0.12	0.25		0.14	0.07	0.37
	10	0.43	0.43	0.05		0.54	0.60	0.44
	11	0.24	0.25	0.21		0.23	0.27	0.45
	12	0.20	0.22	0.27		0.16	0.22	0.59

Passive implant robot								
	No. of implant surgeries	Mandibular left central incisor				Mandibular left first molar		
		Coronal deviation （mm）	Apical deviation （mm）	Axial deviation （°）		Coronal deviation （mm）	Apical deviation （mm）	Axial deviation （°）
Surgeons 2	1	0.28	0.37	0.81		0.35	0.45	0.85
	2	0.60	0.73	1.19		0.53	0.68	1.02
	3	0.55	0.58	0.90		0.73	0.83	0.98
	4	0.71	0.95	1.82		0.43	0.61	1.27
	5	0.16	0.29	1.17		0.34	0.56	1.41
	6	0.44	0.48	0.92		0.33	0.37	0.89
	7	0.28	0.13	0.73		0.44	0.32	1.73
	8	0.19	0.35	0.74		0.21	0.20	0.38
	9	0.27	0.40	0.75		0.26	0.43	1.02
	10	0.41	0.46	0.57		0.47	0.52	0.76
	11	0.62	0.69	1.78		0.26	0.40	1.06
	12	0.47	0.61	0.85		0.38	0.53	1.29

Active implant robot								
	No. of implant surgeries	Mandibular left central incisor				Mandibular left first molar		
		Coronal deviation （mm）	Apical deviation （mm）	Axial deviation （°）		Coronal deviation （mm）	Apical deviation （mm）	Axial deviation （°）
Surgeons 2	1	0.32	0.32	0.64		0.21	0.22	0.63
	2	0.23	0.24	0.47		0.31	0.26	0.45
	3	0.05	0.06	0.42		0.22	0.26	0.64
	4	0.14	0.12	0.40		0.18	0.12	0.64
	5	0.55	0.55	0.47		0.55	0.55	1.04
	6	0.05	0.09	1.09		0.22	0.27	0.51
	7	0.18	0.37	1.00		0.18	0.28	0.64
	8	0.40	0.39	0.49		0.11	0.13	0.40
	9	0.38	0.37	0.75		0.42	0.42	0.36
	10	0.25	0.22	0.97		0.21	0.19	0.42
	11	0.23	0.16	0.62		0.54	0.56	1.00
	12	0.54	0.55	0.55		0.38	0.37	0.09
